# The role of gene expression in ecological speciation

**DOI:** 10.1111/j.1749-6632.2010.05765.x

**Published:** 2010-09

**Authors:** Scott A Pavey, Hélène Collin, Patrik Nosil, Sean M Rogers

**Affiliations:** 1 Department of Biological Sciences, Simon Fraser UniversityBurnaby, BC, Canada; 2 National Park ServiceKatmai National Park, King Salmon, Alaska; 3 Department of Ecology and Evolution, Laboratory for Conservation Biology, University of LausanneLausanne, CH, Switzerland; 4 Institute for Advanced StudyWissenschaftskolleg, Berlin, Germany; 5 Department of Ecology and Evolution, University of ColoradoBoulder, Colorado; 6 Department of Biological Sciences, University of CalgaryCalgary, AB, Canada

**Keywords:** adaptive divergence, eQTL, microarray, phenotypic plasticity, population persistence, qPCR, reproductive isolation, speciation, *cis*-regulatory mutation, gene expression

## Abstract

Ecological speciation is the process by which barriers to gene flow between populations evolve due to adaptive divergence via natural selection. A relatively unexplored area in ecological speciation is the role of gene expression. Gene expression may be associated with ecologically important phenotypes not evident from morphology and play a role during colonization of new environments. Here we review two potential roles of gene expression in ecological speciation: (1) its indirect role in facilitating population persistence and (2) its direct role in contributing to genetically based reproductive isolation. We find indirect evidence that gene expression facilitates population persistence, but direct tests are lacking. We also find clear examples of gene expression having effects on phenotypic traits and adaptive genetic divergence, but links to the evolution of reproductive isolation itself remain indirect. Gene expression during adaptive divergence seems to often involve complex genetic architectures controlled by gene networks, regulatory regions, and “eQTL hotspots.” Nonetheless, we review how approaches for isolating the functional mutations contributing to adaptive divergence are proving to be successful. The study of gene expression has promise for increasing our understanding ecological speciation, particularly when integrative approaches are applied.

## Ecological speciation

Natural selection is a central mechanism of evolutionary change within species. But to what extent is selection also responsible for the formation of new species (i.e., speciation)? Recent years have seen renewed efforts to address this question. Under one scenario, populations living in different ecological environments undergo adaptive genetic differentiation via divergent natural selection, and these same adaptive changes also result in the populations ceasing to exchange genes. Consistent with past work, we define this process of “ecological speciation” as one in which barriers to genetic exchange evolve between populations as a result of ecologically based divergent natural selection.^1^–^5^ Ecological speciation generally occurs because phenotypic traits under divergent selection, or those genetically correlated with them, incidentally affect reproductive isolation.^6^,^7^ Thus, ecological speciation can involve any type of reproductive barrier and can occur under any geographic arrangement of populations (allopatry, parapatry, and sympatry).^1^–^3^,^5^,^8^–^12^ Ecological speciation is distinguished from models of speciation which do not involve ecologically based divergent selection, such as speciation via genetic drift or the fixation of different incompatible mutations in populations experiencing similar selection.^4^,^13^

The process of ecological speciation makes explicit predictions. For example, it predicts that ecologically divergent pairs of populations will exhibit greater reproductive isolation than ecologically similar pairs of populations of similar age.^11^,^14^ Another prediction is that phenotypic traits involved in divergent adaptation will also cause reproductive isolation.^15^ For example, adaptive traits might directly reduce the fitness of immigrants and hybrids, due to a mismatch between immigrant and hybrid phenotypes and the ecological environment, generating “immigrant inviability” and extrinsic postmating isolation, respectively.^16^,^17^ Finally, ecological speciation predicts that neutral gene flow between populations will decrease as adaptive divergence increases.^10^,^18^ These predictions have now been supported numerous times using experiments or molecular data on levels of neutral gene flow (see Refs. 3–5 and 14). Additionally, some progress has been made in understanding the genetic basis of ecological speciation, but this stems primarily from Quantitative Trait Locus (QTL) and candidate gene studies.^4^,^19^,^20^ Here we focus on a largely unexplored issue: the role of gene expression in ecological speciation.

**Figure 1:**
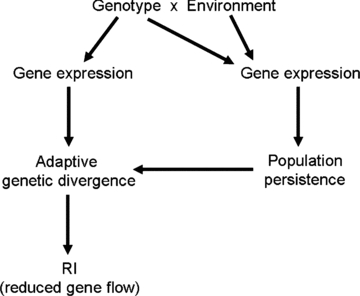
Conceptual diagram of the different ways the genetic and environmental components of gene expression might contribute to ecological speciation. Both components might contribute to population persistence, which is required for eventual speciation. The genetic components of gene expression could contribute to the adaptive genetic divergence, which drives ecological speciation. See text for details.

The role of gene expression warrants consideration because two events need to occur during the process of ecological speciation (following Ref. 21), and gene expression might strongly affect each of them. First, a key mechanism by which ecological divergence between populations occurs is via the colonization of new environments. In these cases, ecological speciation requires that newly founded populations persist in the colonized environments. Ernst Mayr^7^,^8^ especially espoused this “persistence view” of the role of ecology in speciation (for review see Refs. 21, 22). Second, populations in different environments need to evolve genetically based reproductive isolation. Gene expression might therefore promote ecological speciation in two ways: (1) indirectly by promoting population persistence or (2) more directly by affecting adaptive genetic divergence in traits causing reproductive isolation (Fig. 1; Ref. 23).

Here we review both putative roles for gene expression in ecological speciation. Because the study of gene expression and ecological speciation is in its infancy, our goals here are not only to review the existing literature and highlight what is already known, but also to provide a conceptual framework for thinking about the topic and point to especially promising avenues for further research. We begin by providing more detail on why studying gene expression might be fruitful for understanding speciation, followed by a discussion of how to measure gene expression. We then review the roles of gene expression in population persistence and in affecting adaptive genetic divergence.

## What can the study of gene expression tell us about ecological speciation?

Gene expression is shaped by both genetic and environmental components, and can therefore be considered as a “molecular phenotype.”^24^ For example, the transcription rate of a gene can vary among genotypes such that it is a heritable phenotype.^25^–^28^ Gene expression might provide novel insights into speciation because gene expression profiles have the ability to uncover phenotypes, which would not readily be visible via traditional approaches. Our understanding of evolution has often been limited by our ability to define relevant phenotypes.^29^ For example, initial progress in understanding ecological speciation has necessarily focused on easily measured morphological, and to some extent, behavioral traits. In essence, gene expression might allow us to circumvent these limits by uncovering hidden phenotypes potentially of ecological relevance and phenotypes that are perhaps difficult or counter-intuitive to measure. This could be especially critical given that genome annotations to date currently stem mostly from model genetic organisms, and thus are lacking in ecological relevance.^30^ Identifying ecologically relevant expressed genes will thus likely increase the efficacy of genomics to address questions related to ecological speciation.^31^–^33^

For example, physiology has been grossly underrepresented in ecological speciation studies, presumably because of the difficulty associated with measuring these phenotypes.^2^,^5^ With current gene expression technologies, we can now examine many metabolic and mechanistic processes that were previously difficult to measure.^34^,^35^ This may be important because evolutionary changes in expression of physiological genes might sometimes precede morphological changes.^7^ Overall, the sensitivity achievable from modern gene expression technology (large numbers of genes in one assay, low transcript genes, and subtle gene expression differences) has allowed the study of specific organs and tissues and revealed that hidden phenotypes may stem from genes expressed in all of these tissues. For all of these reasons, gene expression studies have the potential for testing numerous hypotheses (i.e., numerous “traits” or genes), many of which an investigator would not necessarily think to test from previous research.^36^ The power of gene expression profiles as surrogate phenotypes is well established in fields such as genetic studies of disease research in humans^29^,^37^ but needs to be further implemented into ecological speciation studies.

Theoretically, this implementation seems possible. Johnson and Porter^38^ demonstrated that parallel directional selection on geographically isolated populations might lead to misregulation of gene expression that in turn may be associated with hybrid incompatibility. By modeling the evolution of a regulated pathway wherein hybrid incompatibility can arise as a consequence of misregulated gene expression, Johnson and Porter^38^ showed that parallel selection is expected to yield reproductive isolation regardless of the underlying mechanisms relating genotype to phenotype. In their analyses, population pairs experienced identical selection conditions and thus did not experience divergent selection. Nonetheless, these results suggest that the detection of gene misregulation may be a feasible starting point towards understanding the role of gene expression in ecological speciation, with the objective of measuring the level of hybrid incompatibility due to gene expression before finding the ultimate mutation responsible.^39^

In summary, gene expression studies may reveal the genes underlying adaptations that are difficult or impossible to measure in other ways, and these phenotypes may be of importance for initiating ecological divergence during speciation.^40^ Consequently, patterns of gene expression should be integrated into studies of ecological speciation, with a need for clearer predictions about how gene expression affects ecological speciation. Gene expression patterns may also provide insight into the underlying genetic architecture of ecological speciation and importantly, if it differs from other types of speciation.

## How to study gene expression

Studies of gene expression measure the expression level of single genes, multiple genes, or the entire transcriptome (the latter defined as all the genes expressed in a cell, tissue or organism). The measure of expression is the abundance of transcribed messenger RNA (mRNA) molecules and is specific to the tissue, developmental stage, point in time, and taxon in which it is measured.^24^,^41^,^42^ Protein and mRNA abundances are highly correlated, that is why mRNA levels can be used as a proxy for differences in protein products.^43^ A variety of methods are now available to quantify gene expression and can be subdivided into two broad categories: (1) those for which (candidate) genes must be known in advance of quantification of expression and (2) those that quantify abundance for multiple genes and thus simultaneously identify genes of interest (Table 1). We treat each category in turn briefly here, and refer readers to previous reviews for greater detail.^44^,^45^

**Table 1:** Comparisons of gene expression methodologies. Technique denoted with (1) do not require *a priori*, whereas techniques denoted with (2) require that candidate genes are known ahead of time

Technique	Approach	Pros	Cons	References
DDRT-PCR (1) Rarely used	A subset of differentially expressed genes	Inexpensive. Generates differentially expressed genes	Only good for genes in high abundance	168
RT-qPCR (2)	Individual candidate genes are compared	Precise, less expensive than some other methods. Great follow-up on transcriptome-wide techniques	Candidate genes are a prerequisite. Primer development can be difficult	169
Microarrays (1)	Up to tens of thousands of genes assayed at one time	Used to generate candidate genes. A large portion of the transcriptome easily screened	Only available for some taxa. Large development cost: expensive	71, 170, 171
SAGE (1)	Many genes assayed and sequenced at one time	Less development cost than microarrays. Does not require functional genome to be sequenced	Fairly expensive but getting cheaper	172, 173
Suppression subtractive hybridization (1) Rarely used	Identify genes differentially expressed	Inexpensive. Generates differentially expressed genes	Require sequencing to identify the physiological function of the differentially expressed genes	54, 55
Northern blot (2)	Probes for a single gene	The original gene expression tool	One gene at a time, limited utility in quantification	46
RNA-Seq via next generation sequencing (1)	Sequence all transcripts	the ultimate tool, price coming down	Expensive, computationally demanding	80–82, 84, 174

We first outline methods that require candidate genes prior to analysis. These are cases where known genes are of *a priori* interest, for example, because of their function or association with ecological variables. The original gene expression technique is Northern blotting where RNA is extracted from a specific tissue and subjected to electrophoresis on a gel.^46^ The gel is then transferred to a nylon membrane that is washed with a labeled probe specific to the candidate gene of interest. If the gene was expressed and the transcript is present, the probe will hybridize and anneal to the membrane. Other samples may then be compared for the expression of this same gene. Northern blots allow the detection and only semiquantification of mRNA target sequences (the darker the band, the greater the expression).^47^ More sensitive techniques have since been developed. One such technique is retrotranscriptase quantitative polymerase chain reaction (RT-qPCR or qPCR).^48^,^49^ With qPCR, one converts mRNA to cDNA and then uses fluorescent probes specific to the cDNA in PCR to monitor the quantity of cDNA template. The PCR cycle associated with exponential growth of product is tightly associated with the quantity of the initial cDNA template, providing an estimate for the level of mRNA expression in the tissue. Depending on experimental design, qPCR can assess relative or absolute abundance of RNA. Since qPCR does not have the same technical problems as microarrays (see below), qPCR has emerged as a method to quantify and verify expression levels of candidate genes identified with large-scale transcriptomic studies.^50^,^51^

A second set of techniques does not require that candidate genes to be chosen prior to analysis. Within this set, two techniques are no longer in common use or have a limited history of use in ecological studies. The first are differential display techniques, which with real-time PCR (DDRT-PCR), can describe differences in gene expression between species.^52^ This strategy is based on the amplification of partial cDNA sequences from a pool of mRNA (of unknown genes being expressed) and is only useful when genes are abundantly expressed. Another method, suppression subtractive hybridization (SSH), employs PCR to differentially amplify cDNA.^53^ SSH has the advantage of identifying all the differentially expressed genes even at low abundance between nonmodel species.^46^ Few studies have used this method to compare gene expression profiles in divergent ecological conditions, presumably because of the availability of more sensitive and precise techniques.^54^,^55^

Currently, the most common technique for extensively assessing global gene expression profiles is microarrays, which are generally akin to a reverse Northern blot.^56^ Microarray experiments are performed by hybridizing “target” cDNA in solution from an experimental group or groups to the spots or “probes” that are fixed to the glass slide, often representing in the order of thousands of genes. Gene expression among groups for each spot are then compared according to their fluorescence intensities to detect up- or downregulated genes. Treatment sample is either competitively hybridized and compared to a common reference or another treatment (two-color experiment) or just the absolute intensity of a single treatment sample is measured (one-color experiment).^57^ The last decade has experienced an explosion of microarray studies in ecology and evolution, and we refer readers to past reviews for a more thorough treatment of the methodology (reviewed in Refs. 42, 49, 58–61).

Here, we focus on two-critical points; susceptibility of type I error and repeatability of the results. First, microarrays are a powerful tool but can be prone to type I errors stemming from the large number of comparisons involved and variation in experimental conditions (e.g., use of different tissues, treatments, ecological types, and species).^41^,^62^ Several methods exist for comparing samples on the arrays,^63^–^65^ along with many different data analysis programs, techniques for normalization, quality control, quantifying spot intensity, and correcting for multiple tests. Second, the repeatability of published microarray studies is arguably limited, especially when the sum of the expression data is unavailable.^66^,^67^ These discrepancies appear to be primarily due to incomplete data annotation or specification of data processing and analysis, rather than technical limitations. Standardized analytical procedures do not exist which can lead to contentious interpretations of the data. Although many journals now require that the data be submitted to acceptable public repositories upon conditional acceptance,^68^ more strict publication rules enforcing public data availability and explicit description of data processing and analysis will be needed to ensure repeatability.

Along these lines, it is important to note that microarrays are intended to be used with the species for which the chip has been developed,^69^ but some studies have demonstrated that microarrays can also be used in closely related species.^49^,^70^,^71^ Oligo microarrays have shorter fragments of cDNA spotted on the chip, so they are less ideal for cross-species work, since few numbers of polymorphism may affect hybridization greatly.^72^ However, cDNA arrays have longer DNA fragments increasing their potential usefulness to nontarget species. For example, the Genomics Research on Atlantic Salmon Project developed a salmonid microarray consisting of expressed sequence tags (ESTs) developed from both rainbow trout (*Oncorhynchus mykiss*) and Atlantic salmon (*Salmo salar*). This microarray has been successfully applied in many closely related salmonids, including other salmon (Salmoninae), whitefish (Coregoninae) and rainbow smelt (*Osmerus mordax*).^73^,^74^ Thus, cDNA microarrays can bridge differences between systems with plentiful genomic resources that are poorly understood ecologically, and systems with a well-known ecology but poorly developed genomic resources.^41^,^75^

An additional technique that assembles many short sequence tags (9–10 bp) excised from cDNA and inserts them in a 1 kbp vector for sequencing is serial analyses of gene expression (SAGE).^76^ Each tag in SAGE can be traced back to a single gene and the relative amount of that tag in the vector corresponds to the mRNA levels in the tissue collected. Thus, SAGE may be used to both identify expressed genes and quantify the relative amounts without any *a priori* ESTs or genomic resources. The latest iteration of this technique, SUPERSAGE assembles longer sequence tags (26 bp) from which primers for qPCR or potentially even oligo microarrays could be created.^77^

With the advent of next-generation sequence techniques, it is now possible to routinely sequence the entire transcriptome of each sample,^78^ even for nonmodel organisms.^79^ Like SAGE, this technique generates sequence data and transcript abundance.^80^,^81^ However, longer sequence reads have the power to discern alternative splice variants,^82^ alternative alleles,^83^ and single nucleotide polymorphisms (SNPs) within coding regions.^84^ This method will therefore be more precise in identifying and quantifying the transcription of closely related genes. As costs decrease and read lengths increase, this may ultimately replace all other transcriptomic methods.

In conclusion, a quickly expanding and improving suite of methods exist for the study of differential gene expression. The best method will depend on the research question, study organism, budget, and whether reasonable candidate genes are known *a priori*. One important gap in the literature is the lack of a comprehensive study comparing the accuracy and precision of these techniques. We now turn to more conceptually oriented questions of how gene expression might affect ecological speciation.

## Gene expression and population persistence

“The creatures which can stand ‘the storm and the stress’ of the physical influences of the environment […] will live; while the others which cannot, will not.”—Baldwin^85^ (1896).

**Figure 2:**
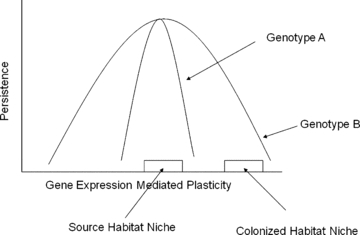
The effects of gene expression-mediated phenotypic plasticity (GMPP: *y*-axis) on colonization of new environments and subsequent population persistence. Genotype A has a lesser breadth of GMPP compared with genotype B. Both genotypes have high persistence in the source habitat, but genotype A has no potential to persist in a colonized habitat. Genotype B's GMPP allows persistence in the colonized habitat, allowing time for adaptive genetic divergence.

The first manner in which gene expression might affect speciation is via promoting population persistence. As exemplified by Baldwin's quotation above, once a population colonizes a new environment, it must persist if it is to speciate. Population establishment and persistence in a new environment may be facilitated by phenotypic plasticity (Fig. 2).^86^–^90^ Modulation of behavioral, morphological, or physiological traits via phenotypic plasticity could therefore occur before any adaptive genetic evolution occurs.^23^ Gene expression-mediated phenotypic plasticity may be described as reaction norms in gene expression with the molecular phenotype of gene expression-facilitating population persistence following colonization.^30^ Direct tests of this idea are lacking, but two lines of indirect evidence exist: (1) studies of plasticity in traits (morphology and behavior mostly) related to fitness and population persistence and (2) studies of gene expression responses during ecological shifts, particularly those resulting in exposure to ecological stress.

First, studies of plasticity in phenotypic traits related to fitness provide evidence for a role for plasticity in population persistence. However, such studies routinely lack evidence on how (or if) gene expression itself was involved. For example, Yeh and Price^88^ studied two populations of dark-eyed Junco birds, a native population in the mountains and a newly established population on the University of California San Diego (UCSD) campus. The UCSD population persisted for years despite significant environmental differences compared to the native habitat. Studies on the length of the breeding season, a classic trait dependent on temperature, revealed that the breeding season of the UCSD populations was twice as long as that of the ancestral populations, presumably due to more favorable climate (e.g., lack of snow) in the newly established population. Importantly, UCSD females displayed higher offspring production without a corresponding increase in mortality, suggesting that plasticity in breeding time was promoting colonization, population establishment, and persistence in the new environment. However, future studies are needed to examine if gene expression might be associated with the shifts in these life history traits.

Second, studies of gene expression response during ecological shifts support a role for gene expression in facilitating responses to ecological change (= stress). By *ecological stress* we mean simply a shift in ecology that affects the fitness of a population. In the last decade, such studies reporting evidence for a role of gene expression during ecological stress have increased (Table 2). Although these studies are critical toward understanding physiological stress response, detailed analysis of the visible phenotypes, and their explicit effects on population persistence, are needed. For instance, McCairns and Bernatchez^91^ examined adaptive divergence between freshwater and marine sticklebacks in a common garden experiment. Specifically, they measured fitness and survival to explore the role of gene expression at four candidate genes in response to osmoregulation. They found a significant correlation between gene expression and fitness and their results thus supported the hypothesis that ancestral plasticity for osmoregulation promoted adaptive divergence via heritable osmoregulation expression (sodium–potassium ATPase). These results are consistent with the hypothesis that gene expression modulation can promote adaptive divergence by allowing populations to persist in a changing environment, whereby fitness is maintained by plasticity.

**Table 2:** Examples of studies showing that gene expression is affected by ecological stress. Under the assumption that gene expression allows populations to better persist in stressful environments, these studies indicate that differential gene expression can promote the colonization of, and subsequent persistence in, novel environments

Organism	Study	Method	Environmental stressor	Proportion of genes affected by treatment	Major physiological function affected
Killfish *Austrofundulus limnaeus*	171	cDNA microarray	Daily and seasonal temperatures regimes	11%	Molecular chaperones, cholesterol and fatty acids synthesis, membrane structure, solute carrier, carbohydrate metabolism, nitrogen metabolism, intermediary metabolism, cytoskeleton elements, protein turnover, complement and innate immunity, and cell growth and proliferation.
Bivalve *Argopecten purpuratus*	175	Suppression subtractive ESTs library + quantitative RT-PCR (candidate genes)	Copper tolerance	8%	Cell differentiation, cellular communication, cytoskeleton, development and differentiation, energetic metabolism, protein regulation, respiratory chain, stress protein, translation and posttranslation processing, cellulose hydrolysis, and ribosomal protein.
Brazilian flounder *Penalichtys orbignyanus*	176	Semi-quantitative RT-PCR (candidate gene approach)	Hyperosmosis	2 candidate genes up-regulated	Growth.
Coral fish *Pomacentrus moluccensis*	177	cDNA microarray	Prolonged heat and hypoxia	2% (down-regulation mostly)	Cell adhesion, cell cycle and growth, cyskeleton, metabolism, protein processing, stress proteins, signal transduction, transcription, translation, and transport.
Arthropod *Orchesella cincta*	27	cDNA microarray	Cadmium	14% (down-regulation mostly)	Translation, signal transduction, stress protein, redox state, general metabolism, chromatin remodeling, and proteolysis digestion.
Antarctic nematode *Plectus murrayi*	178	Suppression subtractive hybridization / ESTs library + quantitative RT-PCR	Desiccation resistance	6%	Carbohydrate metabolism, amino-acid metabolism, lipid metabolism, xenobiotic metabolism, membrane transport, signal transduction, transcription, translation, replication, cell growth and death, and cell communication.
Thale cress *Arabidopsis* spp.	179	Oligo microarray	Salt, osmotic regulation, and temperature	12 to 25% (up-regulation mostly)	Oxidative stress, membrane transport, phosphoregulation, transcription, circadian clock, fatty acid metabolism, stress protein, cytoskeleton, membrane protein, and carbohydrate metabolism.
Black cottonwood *Populus trichocarpa*	180	cDNA micorarray	Herbivory	5% (up-regulation mostly)	Photosynthesis, general metabolism, transport, transcription, octadecanoid and ethylene signaling, detoxification and redox processes, and secondary metabolism.
Atlantic samon *Salmo salar*	110	cDNA microarray	Pathogens	17% (up-regulation mostly)	Immunity-related genes, extracellular matrix component, electron and ion-transport chain, signal transduction, transcription, metal-binding protein, pyrimidine biosynthesis, protein degradation, localization and folding, DNA replication, and cell structure and adhesion.
Mycorrhizal *involutus*	181	cDNA microarray	Fungus *Pillus* Host specificity	16%	Electron transport, lipid and fatty acid metabolism, transcription, sex determination, regulation of cell cycle, glycolysis, stress protein, protein biosynthesis, and aromatic compounds metabolism.

Despite these advances, definitive tests demonstrating that gene expression facilitates population persistence are lacking. Such tests could be carried out using experimental evolution in the lab. For example, the genomics of *Drosophila* is increasingly well characterized, with some mutant lineages able or unable to cope with different stressors.^92^ Under controlled stress conditions, one could measure which genes are most strongly differentially expressed, while controlling for variation in ecologically relevant alternative alleles in different environments.^34^ The differentially expressed genes could then be knocked out in one “expression mutant” treatment (e.g., by the use of RNAi or destroying or inhibiting the promoter regions). Both mutant and control treatments would then be exposed to stress, simulating colonization of a new environment, and the population persistence of each compared. The prediction is that population persistence would be weaker for the mutant treatment. In principle, this experiment could even be conducted in the field.^93^ Similarly, gene expression studies of natural populations colonizing new environments may identify genes and pathways whose plasticity is essential to persistence in new environments.^94^

We note two additional points about the importance of gene expression for population persistence. First, populations and genes with much prestanding genetic variation may exhibit rapid evolution following the colonization of new environments^95^ and thus not require differential gene expression as strongly for population persistence. Second, purely environmentally induced gene expression (nonheritable molecular phenotypes) can still play an indirect role in speciation by facilitating population persistence and “buying the population time” for divergence in other, less plastic, evolvable traits (Fig. 1 in Ref. 23).

## Gene expression and adaptive genetic divergence

The second manner in which gene expression might affect ecological speciation is by being associated with adaptive genetic divergence and reproductive isolation (Fig. 1). This forces a consideration of the link between divergent selection, adaptive genetic divergence, and reproductive isolation: loci under divergent selection and loci causing reproductive isolation are similar in exhibiting reduced introgression (and thus greater divergence) between populations relative to other loci.^16^,^96^–^99^ Indeed, an allele “a” that confers a poor fit of the phenotype to the environment can be selected against and contribute to speciation, whether the afflicted allele resides in one of the parental species (immigrant homozygote “aa”) or in a hybrid individual (heterozygote “Aa”). Recognizing that the adaptive genetic divergence, which results in selection against immigrants and hybrids, represents reproductive isolation itself helps clarify the relatedness of the two processes. Additionally, we stress that adaptive genetic divergence might incidentally cause the evolution of any form of reproductive isolation, including “nonecological” forms such as sexual isolation and intrinsic genetic incompatibilities in hybrids.^5^,^100^

Understanding the heritable component of gene expression will be fundamental toward understanding the genetics of ecological speciation. This is conceptually possible because the expression level for any given transcript is a phenotype that is influenced by both genetics and the environment. The genetic basis of gene transcription itself may exist prior to colonization of new environments or may actually evolve via genetic assimilation.^101^ We consider here two fundamental questions: (1) how substantial is the genetic component of gene expression and (2) can we elucidate whether or not this genetic component of gene expression is associated with adaptive divergence and reproductive isolation? Each question is addressed in a separate section. The main findings, as well as explicit directions for future research, are summarized in Table 3.

**Table 3:** Summary of what is known about gene expression and ecological speciation, what is missing (= future directions), and how these gaps in our knowledge might be addressed

Category/type of study	What is known	What is missing	How to address gaps in our understanding
Population persistence			
1. role of plasticity	Phenotypic plasticity can promote population persistence.	To what extent does this involve gene expression?	Add gene expression data to studies of phenotypic plasticity and population persistence.
2. environmental stress	Gene expression may help populations deal with environmental stress.	Does gene expression during colonization of new environments actually promote population persistence?	Add information on population persistence to studies of gene expression in response to stress.
Heritability of expression divergence			
1. common garden and/or animal model	Expression divergence between populations can have a genetic basis, and can involve parallel evolution across independent populations.	How important is heritable gene expression divergence relative to other forms of genetic divergence (i.e., coding-region changes)?	Integrate studies of gene expression with studies examining functional mutations affecting trait divergence.
2. eQTL	eQTL hotspots exist, exhibit signatures of divergent selection, and provide candidate gene regions for ecological speciation. Networks of gene interactions may be implicated in adaptive divergence.	What role do eQTL hotspots have in adaptive divergence and/or reproductive isolation?	Genome-wide studies will be integral to understanding how gene expression affects ecological speciation.
		To what extent can we establish a mechanistic understanding of gene networks?	
		Can this inform us about the genetics of ecological speciation?	
		Does ecological speciation have a genetic architecture that is different from other types of speciation? If so, why?	
Gene expression and reproductive isolation			
Links between expression, adaptive divergence, and reproductive isolation	Gene expression divergence known to affect adaptive phenotypic divergence, and in some cases has been tied to adaptive genetic divergence. Underlying mutations rarely yet identified.	To what extent does expression divergence actually generate reproductive isolation, either ecologically based or other forms?	Quantify the extent to which expression divergence contributes to reproductive isolation of all forms.
		To what extent can experimental studies of gene expression add to our understanding of the mechanisms of ecological speciation?	
		Can predictions be made about the likelihood of ecological speciation based on gene expression profiles?	
		Is divergence in gene expression associated with the causes of ecological speciation or the consequences?	

## Genetic architecture of gene expression: heritability and eQTL mapping

### Heritability of gene expression divergence

Gene transcription rates can vary among genotypes such that it is a heritable phenotype. Both the magnitude and rate of changes in gene transcription level in response to selection will depend on the heritability of gene expression.^26^,^102^ What proportion of the transcriptional variation in a population is attributable to genetic variation among individuals? Estimation of the heritability of gene expression is likely to be complicated because sources of transcriptional variation can vary tremendously among tissues within individuals, among individuals, and among populations.^103^ Although several studies have discovered gene expression differences between diverging populations, significant transcriptional differences need not reflect heritable genetic variation.^104^

A few studies have formally detected heritable gene transcription differences between populations. These studies quantified genetic differences using common garden experiments, which directly quantify levels of gene expression in the absence of environmental variation.^105^ For example, St-Cyr *et al.* quantified variation in gene expression for almost 4,000 genes in species pairs of lake whitefish from North American lakes under common garden conditions and found that 14% exhibited differences in transcription. These differences are therefore the heritable component of gene expression divergence. Remarkably, genes differentially expressed between species pairs in the common environment were similar to what had been previously identified in the wild. The collective results suggest a predominantly genetic control of differential transcription between these species pairs.^106^

In other studies, heritability of gene expression within a population has been estimated using parent–offspring or sibling regressions. For instance, studies of human gene expression have found that approximately 30% of genes have a significant heritable component.^25^,^107^ Estimating heritability for wild populations is also possible using restricted maximum likelihood (REML) “animal models” applied to multigenerational data from natural populations.^108^ When applied to pedigrees with multiple generations and low immigration rates, these models can reduce bias due to shared environment effects.^109^ Roberge *et al.*^110^ applied this approach to estimate heritability of gene expression in the Atlantic salmon genome, discovering that 16% of 6,500 gene transcripts had a heritable component of gene expression, on average explaining 40% of the variation in transcription profiles. These results compare to other median heritability estimates among genes with heritable transcription profiles ranging from 0.11 (in mice, Ref. 111) to 0.84 (in yeast, Ref. 112). Notably, studies estimating heritability using such approaches need to account for the fact that heritability within a population does not equate to heritable differences between populations. Overall, although there are no studies that have quantified the heritability of transcription profile differences underlying ecological speciation, these results suggest that the heritable component of gene expression exists, but is highly variable.

### eQTL mapping

Analyses on the genetic architecture of transcriptome variation offers to further our understanding of the genetic basis of gene expression and adaptive divergence.^113^ By *genetic architecture* we mean quantifying the number, location, and effect sizes of genes contributing to adaptive divergence.^114^ In studies of genetic architecture, a QTL is defined as a region of the genome containing one or more genes that affect variation in a quantitative trait, identifiable by its linkage or association to polymorphic marker loci.^115^,^116^ Traditional, or “phenotypic” QTL (pQTL) uncover associations between genetic regions and traditional phenotypic traits such as morphology. Expression QTL (eQTL) map transcript abundance in the same manner as pQTL map “traditional” traits. eQTL mapping is emerging as a useful technique for localizing genomic regions contributing to gene expression divergence.^115^ eQTL studies are generally characterized by large numbers of phenotypes (e.g., the number of transcripts on a microarray), but the mapping is typically performed with fewer individuals, due to the still prohibitive cost of running the arrays. Although eQTL studies are still in their infancy, two general patterns have been observed: (1) the predominance of *cis*-localized eQTL and (2) the existence of genomic regions associated with the expression level of many transcripts (so-called eQTL “hotspots”). We consider each in turn.

The segregation of eQTL has a local genomic context because there are two ways, denoted as *cis* or *trans*, that the level of transcript variation may map onto the genome,^116^–^118^ with each providing a different interpretation about genetic architecture. If the transcription profile maps within the gene region for the transcript in question, this association is referred to as *cis* or proximal eQTL. In contrast, if the transcription profile maps to another gene or genomic region it is referred to as a *trans* or distal eQTL.^116^ Cumulatively, the distribution of *cis* versus *trans* eQTL on the genome has shown that *cis* eQTL seem to have larger genetic effect sizes than trans eQTL and that there are more *cis* than *trans* eQTL in the genome,^119^ although the biological interpretation of this pattern remains obscure.^117^,^120^

Another emerging pattern is the existence of eQTL “hotspots”: genomic regions that are associated with the expression level of many transcripts.^26^,^71^ These hotspots may involve the distribution of eQTLs as well as transcriptional covariation between individuals in the mapping family.^113^,^116^ What do these hotspots tell us about the genetics of ecological speciation? First, they show that pQTL and eQTL can map to the same genomic regions. For example, recent studies mapped both eQTL and pQTL for morphological, life-history, and behavioral traits in dwarf and normal lake whitefish species pairs.^19^,^71^,^121^ Of 261 white muscle eQTL distributed over 24 linkage groups, 15 eQTL localized with overlapping pQTL.^121^,^122^ Strikingly, almost 90% of eQTL–pQTL colocalizations involved growth rate and condition factor, two traits central to the adaptive divergence of these species pairs.^19^ Of course, a caveat about overlapping eQTL and pQTLs is that the sizes of the QTL regions are often quite large, such that the apparent colocalization of these two types of QTL need not imply a functional relationship. Nonetheless, the genes within these regions harboring both eQTL and pQTL are arguably strong candidates for genes involved in ecological speciation.

Additionally, eQTL studies indicate that genomic regions involved in ecological speciation can be nonrandomly distributed across the genome. For example, in the same lake whitefish species pairs noted above, 50% of 249 eQTL identified in the brain were associated with only 12 hotspots distributed over eight linkage groups.^71^ A similar pattern was observed in muscle, where 41% of eQTL mapping to six hotspots across four linkage groups.^121^ These findings hint at the existence of localized “genomic islands” of expression divergence, as sometimes reported for islands of genetic differentiation in population genomic studies^123^,^124^ (but see Ref. 125).

Finally, eQTL have also informed us about the actual mechanisms of speciation, for example confirming that mapped genomic regions differentiated via divergent selection. The direction of additive eQTL reported by in the Whiteley *et al.*^71^ and Derome *et al.*^121^ were predominantly in one direction, suggesting a role for directional selection.^2^,^126^ eQTL hotspots have also been associated with molecular signatures of selection in natural populations. For example, in the whitefish species pairs, 10 loci were identified whose genetic divergence in nature exceeds neutral expectations. These are so-called outlier loci subject to divergent selection.^124^,^127^ Three of these outlier loci also corresponded to eQTL hotspots.^122^ Finally, eQTL hotspots may be an indication that coexpression involves a regulatory network such that speciation involves complex interactions between genes.^119^ Overall, eQTL studies can thus be used to infer the genomic distribution of expression profiles^26^ with eQTL distributions potentially informing the mechanisms of gene regulation,^34^ and providing insight into the process of speciation.

### Other approaches to studying heritability of gene expression

The previously discussed approaches to studying the heritability of gene expression divergence may be thought of as top-down or a forward genetics approach: they start with the phenotype or entire transcriptome and work toward narrowing down to regions or genes implicated in adaptive divergence and ecological speciation. However, few QTL maps or genome scans exhibit sufficient resolution to find the exact functional genes or regulatory elements that contain the polymorphisms that are under selection.^127^,^128^ Moreover, mapping approaches may not be feasible in some organisms. Another approach which relies on sequence comparison of functional polymorphisms may be described as a gene expression approach to “reverse ecology”^129^: after differentially expressed functional genes are identified, sequences of the differentially expressed transcripts are compared by direct sequencing efforts that may uncover nonsynonymous mutations in the coding regions of the genes, or genetic polymorphisms in regulatory regions.^130^ Both of these steps may now be accomplished simultaneously with next generation sequencing.^80^ Thus, screening the transcriptome for gene expression differences, even in the absence of a QTL map or genome scan, could simultaneously start the search for functional polymorphisms.

### Genetic component of gene expression: conclusions

Common garden and eQTL studies clearly demonstrate that gene expression divergence can have a heritable component and be associated with adaptive genetic divergence. Although progress has been made in identifying specific differentially expressed genomic regions contributing to adaptive divergence, identification of specific mutations, and characterization of interactions among genomic regions, remains a major challenge for future work.

## Functional links between adaptive candidate gene expression, adaptive genetic divergence, and reproductive isolation

Even after genetic components of gene expression are identified, a major question remains: are these components associated with adaptive divergence and reproductive isolation? Several studies have demonstrated that reductions in hybrid fitness can be due to gene (mis)expression^131^ (for review Ref. 132), in some cases linking gene misexpression in hybrids to other factors previously identified as contributing to ecological speciation.^39^ Along these lines, a growing number of studies have now isolated and characterized specific candidate genes or patterns of gene expression associated with the adaptive divergence which drives ecological speciation (e.g., Refs. 71, 106, 133–135). Of these, none has demonstrated an actual association between gene expression and reproductive isolation (but see Ref. 39), usually because reproductive isolation itself was not explicitly considered, underlying mutations have not been identified, or mutations causing adaptive divergence lie in *cis*-regulatory rather coding regions of genes.^136^,^137^ To compensate, we work under the assumption that genes whose expression is associated with adaptive divergence might also impact the fitness of immigrant and hybrids and thus make a contribution to ecologically based reproductive isolation (“immigrant inviability” and extrinsic postmating isolation), albeit of unknown magnitude. Testing this assumption represents a major avenue for future research. The examples below thus illustrate both the promise and the difficulties associated with linking gene expression to ecological speciation.

### Bmp4: beak shape and speciation in Darwin's finches

**Figure 3:**
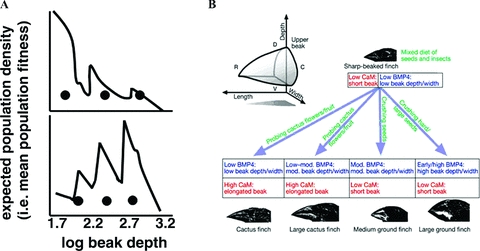
An example of the effects of gene expression in two genes (bone morphogenetic protein 4, *bmp4,* and calmodulin, *CaM*) on phenotypic traits of likely importance for ecological speciation in *Geospiza*, Darwin's finches. (A) Evidence for divergent selection on beak depth from reconstructions of adaptive landscapes. Lines depict the expected population density of a solitary granivorous finch species on two Galápagos islands (similar results were observed on 13 other islands). Dots depict mean log beak depths of actual populations for each curve. Distinct peaks in the adaptive landscape indicate divergent selection, as supported by the observation that actual beak depths differ among populations and tend to correspond to peaks in the landscape. Thus, selection against migrants between environments and intermediate hybrids would likely cause reproductive isolation. Modified from Schluter and Grant^139^ and reprinted with permission of the American Society for Naturalists. (B) Summary of the evidence that *bmp*- and *CaM* -dependent signaling regulates growth along different axes of bill morphology, facilitating the evolution of distinct beak morphologies in Darwin's finches. A beak of the sharp-beaked finch reflects a basal morphology for *Geospiza*. Abbreviations: C, caudal; D, dorsal; R, rostral; V, ventral. Modified from Abzhanov *et al.*^145^ and reprinted with permission of *Nature*.

Darwin's finches arose via adaptive radiation on the Galapagos Islands.^138^ Beak morphology diverged adaptively among populations and species in response to divergent selection stemming from competition and use of seeds of differing size and hardness.^139^,^140^ Beak morphology might also contribute to reproductive isolation via song divergence^141^ or due to selection against immigrants and (intermediate) hybrids.^142^,^143^ Among species, higher levels of the bone morphogenetic protein 4 (*Bmp4*) expression are correlated with deeper beak shapes and over-expression of *Bmp4* in chick embryos altered beak development in the predicted direction.^144^ These results provide compelling evidence that gene expression variation from *Bmp4* affects morphological divergence among species of Darwin's finches (Fig. 3). Similar results occur for another gene, calmodulin (*CaM*^145^). However, due to a lack of common garden or mapping studies, there is as of yet no evidence that heritable differences in beak morphology are affected by *Bmp4* or *CaM*. The mutations underlying beak size differences in Darwin's finches have not been identified. Thus, although there is good evidence that regulatory changes underlie morphological divergence among species of Darwin's finches, the ultimate link between gene expression and genetically based reproductive isolation (= speciation) is yet to be made.

### Pitx1: pelvic reduction and speciation in threespine stickleback

Recently derived postglacial fish populations are among the most extensively studied systems of ecological speciation in nature (reviewed in Refs. 19, 146, 147). One such example is the threespine stickleback (*Gasterosteus aculeatus*) complex in which ecological divergence drove speciation between limnetic and benthic pairs within freshwater lakes, and between marine and freshwater populations.^148^,^149^ Ancestral marine and most derived freshwater stickleback have a robust pelvic apparatus, while at least 24 independent freshwater populations exhibit a greatly reduced or completely absent pelvic structure.^137^,^150^,^151^ Repeated parallel evolution is itself an indication that divergent selection drove evolution, with evidence pointing to predation and differences in ion concentration as the mechanisms of selection.^152^–^155^

Recent studies have examined the genetic basis of pelvic reduction. QTL studies repeatedly identified a single chromosomal region explaining more than two thirds the phenotypic variance in pelvic size.^156^–^158^ Yet, similar to *Bmp*4 in finches, the regulatory mutation contributing to differences in expression remained unknown until recently. Chan *et al.*^137^ reported that a small (501 bp) tissue specific enhancer (*Pel*) drives expression of the gene implicated in pelvic reduction (the *Pitx*1 gene^137^). Remarkably, small deletions functionally inactivated *Pel* in nine of 13 tested pelvic reduced populations. These regions exhibiting recurrent deletions, rather than the *Pitx*1 gene itself, appear to have been subject to positive selection.^137^ These results demonstrate that genetically based expression divergence contributed to adaptive divergence in pelvic morphology. However, direct links to between expression divergence and reproductive isolation remain to be established. The ability to conduct manipulative experiments in seminatural ponds (e.g., Refs. 95, 159) indicates that linking gene expression at *Pitx*1 to reproductive isolation (i.e., reduced fitness of immigrants and hybrids) is a distinct possibility.

### Other examples

There are many other examples of studies of gene expression and adaptation, but few make links to adaptive genetic divergence, and thus few pertain directly to ecological speciation. For instance, cichlid fish species have adapted to divergent light environments within lakes, via the effects of gene expression on the tuning of visual perceptual sensitivity.^160^ In this case, changes in gene expression contribute to sensory diversification in replicate radiations of cichlid fishes in the clear waters of Lake Malawi versus the turbid waters of Lake Victoria, and functional substitutions contributing to expression divergence were identified.^161^ These studies demonstrate important findings with respect to the molecular basis of ecologically driven sensory diversification, but again a direct demonstration that this contributed to reproductive isolation does not yet exist.

Mimetic wing coloration in *Heliconius* butterflies gives rise to wing patterns that show repeated convergence between species and have adaptive value in mimicry and mate choice, thus potentially associated with ecological speciation.^162^–^165^ Comparative gene expression between two species, *H. erato* and *H. melpomeme*, found that *cinnabar* expression correlated with the forewing band, providing good evidence that the expression of this gene gives rise to the red-banded phenotype in both species.^162^ Chamberlain *et al.*^166^ report similar associations between wing color and gene expression, but within polymorphic populations. Differences in the actual traits in these studies (wing color and pattern) are heritable, but once again functional mutations contributing to reproductive isolation are lacking.

On the other hand, recent genome-wide analyses of the transcriptome have demonstrated that complex patterns of gene misexpression may underlie reproductive isolation mechanisms in hybrids. Renault *et al.*^39^ contrasted gene expression divergence at key early developmental stages in species pairs of normal and dwarf whitefish (*Coregonus clupeaformis*) and their F1 hybrids to identify the main mode of action responsible for gene transcription and to discover key genes misexpressed in hybrids. Although only five of 5,000 transcripts differed in mean expression level between parentals and hybrids at the embryonic stage, 617 out of 5,300 transcripts differed significantly for 16-week-old juveniles. Remarkably, significant gene misexpression in backcross hybrids involved several genes, most notably the disruption of three key developmental genes involved in protein folding and mRNA translation. Overall, direct demonstrations of how gene expression causes reproductive isolation remains a major missing link in connecting the role of gene expression to ecological speciation. Once such demonstrations are made, it will be necessary to test whether, and how, expression divergence actually reduces gene flow between natural populations.

## Conclusions and future directions

Gene expression is likely to be important for the two events required for ecological speciation: population persistence and the evolution of genetically based reproductive isolation. Studies of plasticity and population persistence have yet to address gene expression explicitly. When it comes to adaptive genetic divergence and reproductive isolation, gene expression divergence has been shown to be heritable and to contribute to adaptive genetic divergence, but links to the evolution of reproductive isolation remain indirect (see Table 3 for a summary of what is known, and what needs to be done next). Our review suggests that establishing this link will be challenging because the genetic architecture of ecological speciation can be controlled by gene networks and regulatory regions, rendering an understanding of the functional association between gene expression and adaptive divergence difficult. This implies that it may be difficult to make predictions about the likelihood of ecological speciation based on gene expression profiles until we have a better idea about the genetic architecture of ecological speciation and how it compares to other mechanisms of speciation.^19^,^99^ Nonetheless, isolating the mutations contributing to variation in adaptive traits, and then studying their effects on reproductive isolation, is a necessary task for understanding how gene expression affects ecological speciation.^137^ This is also important for establishing whether gene expression changes are associated with the causes of ecological speciation, or are the consequences. Such goals will likely be best achieved by integrating multiple molecular techniques with experimental studies of how different mutations (alleles) affect fitness and reproductive isolation.^4^,^34^,^95^,^167^
